# Commentary: Prenatal Ethanol Exposure Persistently Alters Endocannabinoid Signaling and Endocannabinioid-Mediated Excitatory Synaptic Plasticity in Ventral Tegmental Area Dopamine Neurons

**DOI:** 10.3389/fnmol.2017.00364

**Published:** 2017-11-03

**Authors:** Hada Fong-ha Ieong

**Affiliations:** Faculty of Health Sciences, University of Macau, Taipa, China

**Keywords:** eCB system, positive reinforcement, addiction, reward system, mGluR, glutamate, cannabinoid receptors, early adversity

The endocannabinoid (eCB) system (ECS) plays a prominent role in regulating brain reward function and modulating emotional homeostasis (Parsons and Hurd, 2015; Araque et al., 2017) and stress (Volkow et al., 2017). Adverse effects on the ECS during early development have been hypothesized to increase the likelihood of future engagement in drug use (Koob and Le Moal, 2008), characterized by positive reinforcement (Wise and Koob, 2014). This reinforcement driving drug-seeking behaviors is thought to result from the imbalance in glutamate homeostasis that disrupts the mesocorticolimbic dopamine (DA) pathways (Kalivas, 2009). Among many adverse factors during distinct developmental stages, however, the molecular underpinnings of the transition to addiction remain largely elusive. A better understanding of synaptic plasticity underlying the ECS associated with early adversity and addiction risk is important to guide the development of future therapeutic interventions (Parsons and Hurd, 2015).

Plasticity of glutamatergic synapses in the ventral tegmental area (VTA) has been implicated as a critical component of long-lasting neural maladaptation underlying addictive behaviors (Kauer and Malenka, 2007). Prenatal ethanol (PE) exposure has shown increased addiction risk in literature (Abel et al., 1981; Baer et al., 2003; Koob and Le Moal, 2008). Expanding this line of research, a recent work (Hausknecht et al., 2017) discovered that PE exposure persistently impaired eCB long-term depression (LTD) at excitatory synapses in rats.

Three mechanisms by which the eCB-LTD at VTA DA synapses can be altered are changes in presynaptic receptor and postsynaptic receptor levels and eCB biosynthesis. These mechanisms have been consistently implicated in neuronal maladaptation accompanying ethanol exposure in adult rodents. For example, the presynaptic cannabinoid 1 (CB1) receptors were downregulated by chronic ethanol administration (Basavarajappa and Hungund, 1999; Xia et al., 2006; Mitrirattanakul et al., 2007; DePoy et al., 2013; Varodayan et al., 2016). eCB-LTD in midbrain DA neurons was mediated by reduced presynaptic glutamate release (Haj-Dahmane and Shen, 2010). Moreover, increased eCB ligand 2-arachidonoylglycerol (2-AG) level was observed in nucleus accumben (NAc) and hippocampus after acute and chronic ethanol administration (Caillé et al., 2007; Mitrirattanakul et al., 2007). Considering that the effects of acute and chronic ethanol exposures in mature animals are relatively temporary, are these mechanisms also at work in PE exposure, in which its effects tend to have long-term consequences?

To assess the change in presynaptic CB1 receptor levels and its role in the PE-induced impairment of eCB-LTD, Hausknecht et al. (2017) used a CB1 receptor agonist to inhibit the evoked excitatory postsynaptic currents (EPSCs) and examined the PE effects on inhibition. They found that CB1 receptors were reduced, and it was associated with the impaired eCB-LTD in PE rats (Hausknecht et al., 2017, their Figure 3A). They also used a CB1 receptor antagonist/inverse agonist to confirm the exhibited inhibitory effect mediated by CB1 receptor activation. Their findings showed that downregulation of CB1 receptors impaired eCB-LTD and suggested that PE exposure might mediate the downregulation. Interesting questions are raised. First, are the effects of PE exposure specific to excitatory synapses in VTA? There is a notion that chronic ethanol exposure in adult animals alters inhibitory GABAergic synapses in striatum (Melis et al., 2002; Adermark et al., 2011). It is unknown if PE exposure influences the excitatory synapse selectively, or if it affects both of the excitatory and inhibitory synapses in an equivalent manner. Second, what are the mechanisms underlying the long-term eCB-LTD impairment after the prenatal insult?

Dysregulation of ligand synthesis may also contribute to the impaired LTD mechanism. Hausknecht et al. (2017) found that, using a potent agonist of postsynaptic type 1 metabotropic glutamate (mGlu) receptors, the LTD was induced in both PE and control animals, and using a CB1 receptor antagonist/inverse agonist, the induced LTD was blocked (Hausknecht et al., 2017; their Figure 7). They further highlighted that, in PE rats, activation of mGlu receptors rescued the eCB-LTD but increasing glutamate level alone did not. Two key mechanisms are raised: (1) eCB ligand synthesis in VTA DA neurons and its release to synapses mediate eCB-LTD, consistent with previous evidence (Parsons and Hurd, 2015; Araque et al., 2017), and (2) the persistent effect of PE exposure on the eCB-LTD at excitatory synapses and the downregulation of CB1 receptors are the primary cause of the diminished eCB-LTD. Another interesting question is then raised: what is the underlying cellular mechanism when the LTD is rescued in PE animals? Because both presynaptic glutamate stimulation and postsynaptic membrane depolarization are necessary to induce eCB-LTD (Haj-Dahmane and Shen, 2010), one would assume that the eCB synthesis and CB1 receptor function are intact. However, under PE condition, it is unclear how the LTD is rescued by mGlu receptor activation when CB1 receptors are downregulated. Would it be because of the variations in eCB and synthetic agonist actions and/or changes in the receptors? In either case, it should be noted that the CB1 agonist function, or the efficacy of CB1 receptors, may be intervened by receptor levels, receptor internalization, and/or uncoupling from the subunits of G proteins without changes in protein expression.

The source of eCB signaling (e.g., tonic and phasic activities) and its application to other drugs of dependence or other prenatal stresses remain to be determined. Moreover, the extent of the excitatory pathway linked to the widely-studied inhibitory GABAergic pathway on the VTA DA neurons should be investigated. The CB1 receptors examined by Hausknecht et al. (2017) were at excitatory synapses, but these G protein-coupled receptors are the most abundant at inhibitory synapses in adult brain. The interactions between these two pathways and eCB-LTD may yield an insight on the transition to drug addiction in a living and dynamic environment including humans. Other variables, such as, choice of species, genetic makeup, eCB clearance, drug dose used, duration of treatment, and sex differences, could also affect the mechanism underlying the molecular impairments and behavioral responses. Such models are essential to comprehensively appreciate the contribution of receptors signaling to positive-reinforcing responses and to evaluate therapeutic potential for normalizing eCB signaling by activating mGlu receptors and/or restoring CB1 receptor function.

These considerations notwithstanding, the observations of Hausknecht et al. (2017) advance our understanding of the synaptic foundations of impaired eCB-mediated plasticity of excitatory signaling in VTA DA neurons associated with PE exposure. Their work (Hausknecht et al., 2015, 2017) facilitates further investigation of other functionally accordant aspects of VTA excitatory synaptic plasticity (e.g., changes in spine head morphology, directional regulation, CB1/CB2 binding ratio). Considering the crucial role PE exposure plays in profoundly altering eCB signaling and excitatory molecular plasticity in VTA and increasing risk in addiction, the findings of Hausknecht et al. (2017) raise the possibility of using mGlu receptor agonists to rescue the eCB-LTD (Figure 1) and to ameliorate addiction risk in PE.

**Figure 1:**
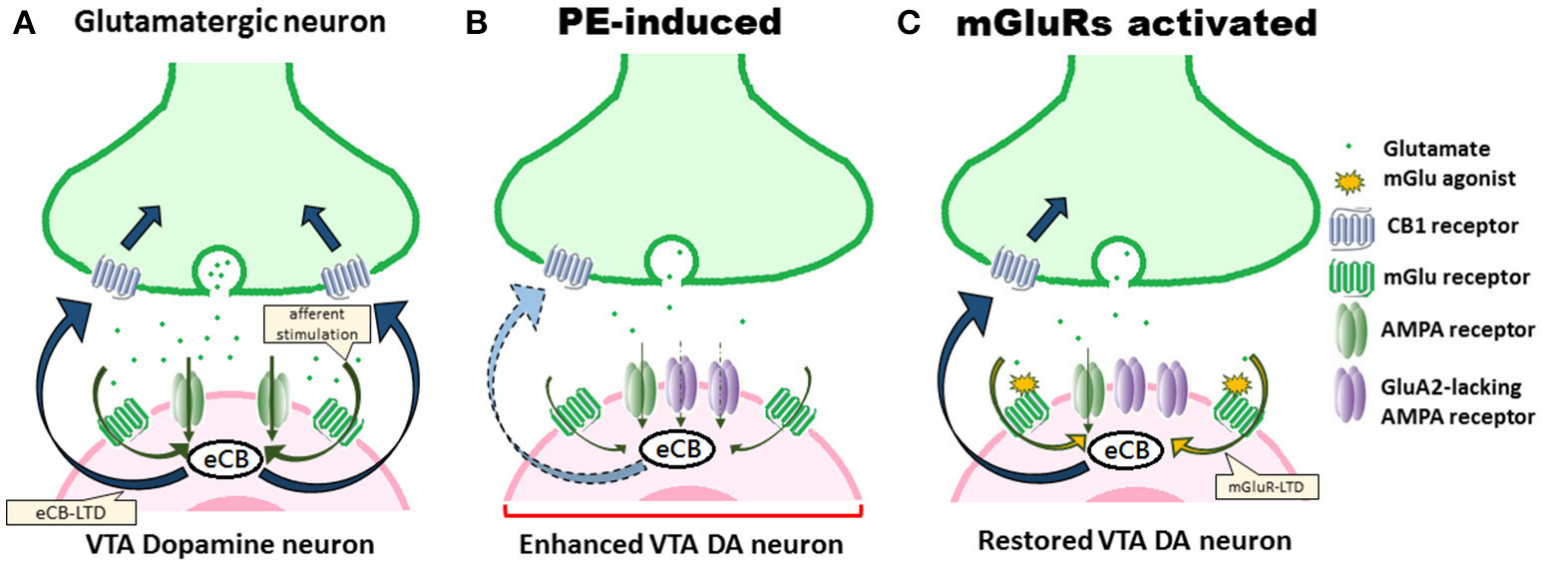
PE-induced alterations of eCB signaling and excitatory synaptic plasticity in VTA DA neuron underlie positive reinforcement. **(A)** Under normal conditions, eCB-LTD is induced by afferent stimulation, resulting in eCB synthesis. eCBs then activate CB1 receptors. **(B)** PE exposure persistently alters eCB signaling, impairs eCB-LTD, downregulates CB1 receptors, and thus results in the enhanced excitability of VTA DA neurons that underlie positive reinforcement states that promote addiction-related behaviors. Upregulation of AMPA receptors, including the polyamine-lacking receptors, and the decreased glutamate stimulation further contribute to an occlusion effect on the eCB signaling. **(C)** Drug-induced adaptations can be counteracted by using mGlu receptor agonists, signaling eCB production, leading to rescue of eCB-LTD and restoration of augmented excitatory synaptic strength in VTA DA neurons.

## Author contributions

The author confirms being the sole contributor of this work and approved it for publication.

### Conflict of interest statement

The author declares that the research was conducted in the absence of any commercial or financial relationships that could be construed as a potential conflict of interest.
